# A central role for MeCP2 in the epigenetic repression of miR-200c during epithelial-to-mesenchymal transition of glioma

**DOI:** 10.1186/s13046-019-1341-6

**Published:** 2019-08-20

**Authors:** Erbao Bian, Xueran Chen, Yadi Xu, Xinghu Ji, Meng Cheng, Hongliang Wang, Zhiyou Fang, Bing Zhao

**Affiliations:** 1 Department of Neurosurgery, The Second Affiliated Hospital of Anhui Medical University, 678 Fu Rong Road, Hefei, 230601 Anhui Province China; 2 Cerebral Vascular Disease Research Center, Anhui Medical University, Hefei, 230601 China; 3 Anhui Province Key Laboratory of Medical Physics and Technology, Center of Medical Physics and Technology, Hefei Institutes of Physical Science, Chinese Academy of Sciences, No. 350, Shushan Hu Road, Hefei, 230031 Anhui China

**Keywords:** Glioma, MeCP2, miR-200c, SUV39H1

## Abstract

**Background:**

The epithelial-to-mesenchymal transition (EMT) has been linked to the regulation of glioma progression. However, the underlying signaling mechanisms that regulate EMT are poorly understood.

**Methods:**

Quantitative real-time PCR (RT-qPCR) and western blot were performed to detect the expression of MeCP2 in glioma tissues and cell lines. MeCP2 functions were tested with cell immunofluorescence staining and western blot. For in vivo experiments, mouse xenograft model was used to investigate the effects of MeCP2 on glioma. ChIP and Co-IP were used to detect the relationships among MeCP2, miR-200c and Suv39H1.

**Results:**

In this study, we found that MeCP2 was frequently up-regulated in human glioma tissues and cell lines. MeCP2 knockdown remarkably induced cell epithelial phenotype and inhibited mesenchymal marker ZEB1 and ZEB2 in vitro and in vivo. In addition, MeCP2 in glioma tissues was negatively correlated with miR-200c expression, and miR-200c overexpression partially abrogated mesenchymal phenotype induced by MeCP2. More importantly, we showed that MeCP2 recruited H3K9 to the promoter of miR-200c by interacting with SUV39H1, resulting in EMT of glioma cells.

**Conclusions:**

This study for the first time reveals MeCP2 as a novel regulator of EMT in glioma and suggest that MeCP2 inhibition may represent a promising therapeutic option for suppressing EMT in glioma.

**Electronic supplementary material:**

The online version of this article (10.1186/s13046-019-1341-6) contains supplementary material, which is available to authorized users.

## Background

Gliomas are the most common primary brain tumor characterized by highly infiltrative growth. Base on the pathological characteristics, gliomas can be classified into four clinical grades. (Glioblastoma multiforme, GBM) is one of the most aggressive types of brain tumors, and despite the combination of multiple treatments, including surgery, chemotherapy and radiation, patients often still develop refractory recurrence [1]. In general, GBM patients have a median survival time of no more than 16 months after optimal treatment [2]. GBM are classified into four molecular subtypes including mesenchymal, classical, proneural and neural subtypes based on gene expression-based molecular classification [3].The mesenchymal GBM subtype has recently been shown to be the most malignant with resistance to radiotherapy and chemotherapy. This pathogenic phenotype has been associated with the epithelial-to-mesenchymal transition (EMT). The EMT is a key biological process that is normally involved in embryonic development and have been reported to regulate invasion and metastasis of tumor [4, 5]. In GBM, members of the ZEB-family, e.g., ZEB1 and ZEB2, known as the activators of EMT, can promote the invasiveness of GBM cells [6, 7]. Therefore, understanding the molecular mechanism of EMT is essential for the development of novel and effective therapeutic strategies for gliomas.

Methyl CpG-binding protein 2 (MeCP2) is a member of the methyl-CpG-binding domain (MBD) family of proteins [8]. MeCP2 has been found to have two functional domains, a 104-amino-acid transcriptional repression domain (TRD) and an 85-amino-acid MBD. MBD binds DNA sequences methylated at cytosine in the dinucleotide 5′-CpG and TRD acts as a transcriptional repressor by recruiting histone deacetylase complex (HDAC) [9, 10]. MeCP2 has been reported to be implicated in a number of molecular functions, such as transcription regulation, RNA splicing, and chromatin organization [11, 12]. Loss-of-function mutations in MeCP2 causes Rett syndrome (RTT), whereas the duplications of MeCP2-containing loci may result in spectrum of phenotypes ranging from autism to intellectual disabilities and mood disorders [13, 14]. Recently, it has been reported that a non-neuronal role for MeCP2 has emerged in tumorigenesis, such as prostate cancer, breast cancer and gastric cancer [15, 16]. However, little is known about its biological characteristics and molecular mechanisms in human glioma.

MicroRNAs, known as small noncoding RNAs, are involved in regulation of downstream gene expression at the posttranscriptional level [17]. Most importantly, the complex regulatory network not only allows one gene by the combination of multiple microRNAs, but also modulates the expression of several genes via one microRNA [18, 19]. Recently, numerous deregulated miRNAs have been reported to be associated with human cancers progression [20, 21]. The miR-200 family (miR-200c/miR-141 and miR-200a/miR-200b/miR-429 clusters) is a tumor-suppressive group of miRNAs that is implicated in suppressing EMT process [22, 23]. Recent reports have suggested that the repression of miR-200 s expression occurs due to epigenetic modification, such as DNA methylation and histone methylation [24–26]. In addition, our previous study showed that miR-141 methylation mediated by DNMT1 in glioma. However, the association of MeCP2 with miR-200 s dysregulation is still unknown.

In this paper, we found that MeCP2 knockdown repressed EMT process of glioma cells, and expression of ZEB1 and ZEB2. Additionally, MeCP2 regulated the expression of microRNA-200 family targeting ZEB1 and ZEB2 transcripts through epigenetic modification. The present work reveals epigenetic regulatory network between miRNA and methylation, which will provide a novel therapy strategy for guarding against EMT of gliomas via targeting MeCP2 and its downstream miR-200c.

## Materials and methods

### Patients and tissue samples

Resected brain tumors were collected from the Department of Neurosurgery of The Second Affiliated Hospital of Anhui Medical University (Hefei, China) after obtaining all patient’s or their client’s informed consent. Tissue samples included 12 normal brain tissues and 65 glioma tissues. All cases were pathologically graded as low grade (WHO I/II, *n* = 22) and high grade (WHO III/IV, *n* = 43) according to the WHO criteria, and the information about the essential characteristics of these tumors was placed in Additional file 1: Table S1. Samples were preserved in liquid nitrogen and stored in − 80 °C. This experiment was approved by the Research Ethics Committee of The Second Affiliated Hospital of Anhui Medical University.

### TCGA data assay

The Cancer Genome Atlas (TCGA) assay was carried out by website http://cancergenome.nih.gov/ as previous described [27]. This TCGA cohort include normal tissues (*n* = 207), low grade (*n* = 518) including all low-grade gliomas, and GBM (*n* = 163), and genes expression were analyzed. In addition, GBM subtypes expression data according to their molecular classification (classic, proneural, neural, mesenchymal) were obtained and the relationship between the expression of genes in glioma was analyzed by in this database.

### Cell culture

Glioma cell lines (U251, LN18, A172, and U87) and normal human astrocytes (NHAs) were obtained from the Cell Bank of the Chinese Academy of Sciences (Shanghai, China) and Sun Yat-Sen University, respectively. Cells were grown in DMEM and supplemented with 10% fetal bovine serum (FBS, Gibco, USA), 100 U/ml penicillin/streptomycin (Sigma), in the presence of 5% CO_2_ at 37 °C.

### Immunofluorescence staining

Cells were inoculated into the 6-well plate comprising slide and cultured overnight. After that, cells were fixed with 4% paraformaldehyde (Beyotime Biotechnology, ST476). Cells were permeated with 0.5% Triton X-100 (Beyotime Biotechnology, ST795) in PBS and blocked with 5% bovine serum albumin (BOSTER, AR0004) and incubated with β-tubulin (cell signaling,cat#2146), ZEB1 (Abcam, ab245283) and ZEB2 (Abcam,ab138222) antibody overnight at 4 °C.FITC conjugated secondary antibody was used to detect primary antibody, and then DAPI was used to incubate cells for counterstaining. Images were acquired using a DP71 fluorescence microscope with a digital color camera (Olympus, USA).

### Cell transfection

The human MeCP2-WT gene and other constructs of MeCP2 truncations MeCP2^ΔN^, and MeCP2^ΔC^ were generated according to manufacturer’s instructions. Sh-MeCP2 (5′-TGCTTAAGCAAAGGAAATCTCTCGAGAGATTTCCTTTGCTTAAGCTTTTTTC-3′) was provided by GenePharma (Shanghai, China), and its corresponding non-targeting sequence (sh-control). si-SUV39H1 and nonspecific control siRNA (si-NC) were synthesized (Genepharma, Shanghai, China). Plasmid was transfected into U251 and LN18 glioma cells respectively by using Lipofectamine 2000 (Invitrogen, USA) according to the manufacturer’s protocol. MiR-200c mimics and miR-200c negative control (NC) were purchased from RiboBio (Guangzhou, China), and transfected into cell lines according to the manufacturer’s protocol.

### RNA extraction and quantitative real-time PCR

Total RNAs from tissues and cultured cells were extracted using Trizo reagent (Invitrogen, USA) according to the manufacturer’s protocol. cDNA was reversely transcribed by using PrimeScriptTM RT Master Mix (Perfect Real Time) (TaKaRa Biotechnology, China). All-in-One™ miRNA First-Strand cDNA Synthesis Kit (Genecopoeia, China) was used for miRNA reverse transcription and RT-qPCR was performed using All-in-One™ miRNA qPCR Kit (Genecopoeia, Guangzhou, China) of miR-200a (Cat#HmiRQP0298), miR-200b (Cat#HmiRQP0300), miR-200c (Cat#HmiRQP0302), miR-141(Cat#HmiRQP0184), miR-429 (Cat#HmiRQP0497) and U6 (Cat#HmiRQP9001). Real-time PCR was performed with SYBR Green detection chemistry (TaKaRa Biotechnology, China) on ABI 7500 Real-Time PCR System (Applied Biosystems). For relative quantification, 2^-ΔΔCt^ was calculated, with U6 RNA as a reference in miRNA analysis and GAPDH as a reference in the analysis of protein coding genes. The indicated primers were placed in Additional file 2: Table S2.

### Western blotting

Tissues and cells were treated with RIPA lysis buffer (Beyotime, China). Total protein extracts (20 or 40 μg) were subjected to 8% or 12% SDS PAGE separation and then transferred to PVDF membrane (MilliporeCorp, U.S.A.). Membranes were probed with targeted primary antibodies: Anti-β-actin (Abcam, ab179467), Anti-MeCP2 (Abcam, ab195393), anti-ZEB1 (Abcam, ab203829) and anti-ZEB2 (Abcam, ab138222). Following blots were washed three times in TBS/Tween-20 and then incubated with appropriate horseradish peroxidase (HRP)-conjugated secondary antibody at a 1:10000 dilution in TBS/Tween-20 containing 5% milk. Proteins were visualized using an ECL chemiluminescence kit (ECL-plus, Thermo Scientific).

### Co-immunoprecipitation (co-IP) assay

Co-IP assays were performed to examine the interaction between MeCP2 and SUV39H1 according to the manufacturer’s protocol, and complexes were precipitated with protein A/G agarose (Bimake, B23201). Then, complexes were subjected to western blot analysis using anti-MeCP2 (Abcam, ab195393), anti-SUV39H1 (Abcam, ab12405).

### Chromatin immunoprecipitation (ChIP)

ChIP assays were performed according to previously described Protocols [28]. In brief, ChIP was performed using the EZ-Magna ChIP Chromatin Immunoprecipitation Kit (Millipore). The antibodies were obtained from Abcam: anti-H3K9 (ab8898) and anti-SUV39H1 (Abcam, ab12405). The DNA was detected through RT-qPCR and primers were provided in Additional file 2: Table S2.

### Tumor formation study in vivo

All mouse experiments were approved by the Animal Research Committee of Anhui Medical University. LN-18 glioma cells infected with lentiviral vectors (GeneChemCo.Ltd., Shanghai, China) containing sh-MeCP2 and sh-con were suspended in 200 μl PBS and subcutaneously injected in BALB/c female nude mice (5 × 10^6^cells/mouse).Tumor size was measured every 7 days using an electronic caliper, and the tumor volume was determined with the formula:V = 0.5 × L (length) × W^2^ (width) [29]. Mice were sacrificed after 6 weeks at cell inoculation, and tumors were excised and evaluated for volume.

### Immunohistochemistry

Samples were fixed in 4% paraformaldehyde, and then were dehydrated, embedded in paraffin, and sectioned. Immunohistochemistry staining was performed according to the manufacture’s protocol using antibodies against ZEB1 and ZEB2 (Abcam, USA). Images were visualized using florescence microscope. Results of immunohistochemical (IHC) staining was analyzed by Image proPlus6.0.

### Statistical analysis

Data are presented as mean ± SD of three independent experiments, in which each assay was performed in triplicate. Statistical analysis was performed using the GraphPad Prism 5 software. The correlation between the expression of MeCP2, miR-200c, ZEB1 and ZEB2 in tissues was analyzed with Pearson’s correlation. A *p*-value < 0.05 was considered statistically significant.

## Results

### Increased MeCP2 levels in gliomas tissues and cells

**Fig. 1:**
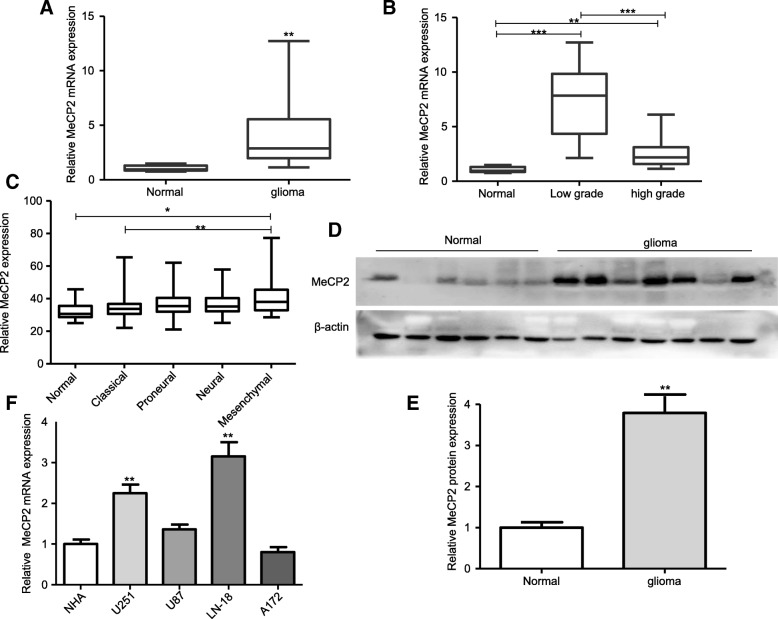
The upregulation of MeCP2 expression in gliomas. **a** The levels of MeCP2 mRNA expression was increased in glioma samples. **p < 0.01 vs. Normal. **b** Increased malignancy of glioma was associated with increased MeCP2 mRNA expression. ***p < 0.001, **p < 0.001 vs. Normal, ***p < 0.001 vs. Low grade. **c** MeCP2 is strongly increased in the mesenchymal subgroup of glioblastoma. *p < 0.05 vs. Normal, **p < 0.01 vs. Classical. **d**-**e** The levels of MeCP2 protein expression in normal brain and glioma tissues. **p < 0.01 vs. Normal. **f** The level of MeCP2 in glioma cell lines and normal human astrocytes (NHA). **p < 0.01 vs. NHA

To evaluate MeCP2 expression in glioma samples and cell lines, MeCP2 expression were detected by RT-qPCR. As shown in Fig. 1a, MeCP2 mRNA expression was markedly increased in glioma tissues compared with normal brain tissues. To determine whether the expression of MeCP2 is linked to the pathogenesis of glioma, MeCP2 expression in glioma tissues with different histopathologic grades was observed. A significant increase was observed in low grade glioma samples, whereas a slight increase in high grade glioma samples (Fig. 1b). Interestingly, MeCP2 expression is significantly increased in mesenchymal glioblastoma as compared with classical subgroup as divided after Verhaak et al. [3] (Fig. 1c). To confirm the changes of MeCP2 expression in gliomas, immunoblotting analysis was performed. MeCP2 protein expression was increased in glioma tissues compared with that in normal brain tissues (Fig. 1d-e). Moreover, we also measured MeCP2 levels in a panel of glioma cell lines and a normal human astrocytes (NHA). Compared with NHA, MeCP2 expression levels were significantly increased in LN-18 and U251 glioma cell lines (Fig. 1f). Therefore, we chose LN-18 and U251 glioma cells for further functional studies.

### MeCP2 knockdown induces epithelial phenotype in vitro

**Fig. 2:**
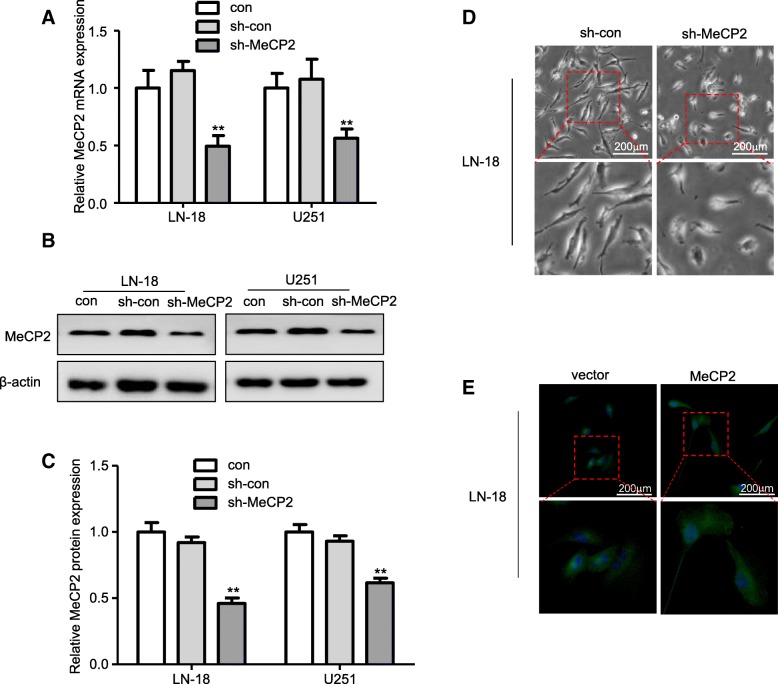
MeCP2 knockdown induced epithelial phenotype in glioma cells. **a** The levels of MeCP2 mRNA expression were examined after transfection with MeCP2 shRNA. **p < 0.01 vs. sh-con. **b**-**c** The levels of MeCP2 protein expression were examined after transfection with MeCP2 shRNA. **p < 0.01 vs. sh-con. **d** LN-18 glioma cell morphology was captured with optical microscope after transfection with MeCP2 shRNA for 2 weeks. **e** Representative images of LN-18 cell morphology were captured after transfection with MeCP2 plasmid. Green, β-tubulin. Blue, DAPI for nucleus

**Fig. 3:**
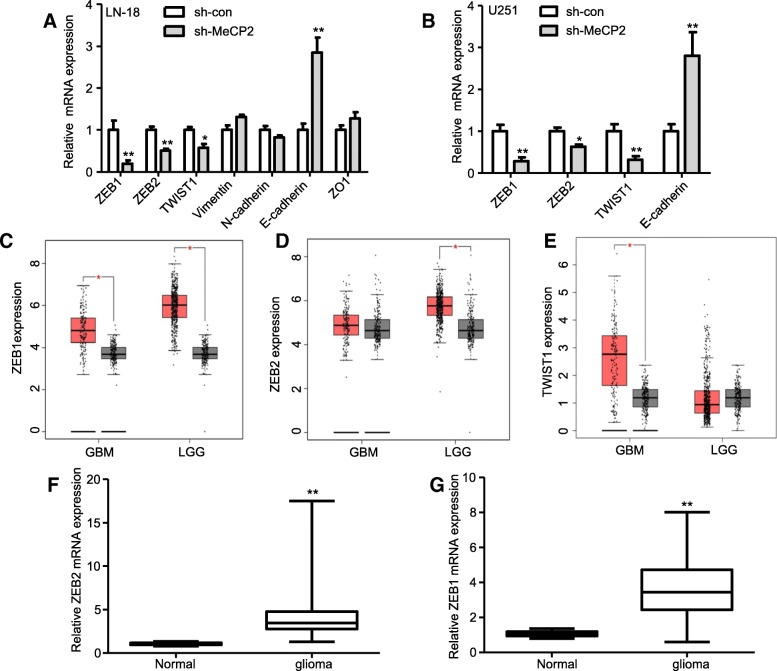
MeCP2 regulated EMT genes in glioma. **a**-**b** The levels of mRNA EMT genes in glioma cells transfected with MeCP2 shRNA. *p < 0.05, **p < 0.01 vs. sh-con. **c**-**e** The level of EMT genes ZEB1, ZEB2 and TWIST1 in glioma and normal tissues samples from TCGA cohort. Red, tumor. Black, normal. This TCGA cohort include normal tissues(*n* = 207), low grade (LGG, *n* = 518) and GBM (*n* = 163). **f**-**g** The levels of ZEB1 and ZEB2 in glioma and normal tissues samples. **p < 0.01 vs. Normal

To investigate the effect of MeCP2 on EMT in gliomas, LN-18 and U251 glioma cells were transfected sh-MeCP2 and sh-con. Decreased expression of MeCP2 after transfection of sh-MeCP2 was confirmed in LN-18 and U251 glioma cell lines (Fig. 2a-c). In addition, obvious cell morphology change was observed in the second weeks after MeCP2 knockdown, and cells morphology were changed from mesenchymal morphology to epithelial morphology (Fig. 2d and Additional file 3: Fig. S1a). Staining microtubules by β-tubulin antibody was used to detect the morphological changes. As shown in Fig. 2e and Additional file 3: Fig. S1b-c, MeCP2 overexpression increased spindle shaped morphology compared with vector. To determine whether MeCP2 regulate EMT in glioma, the expression of EMT markers was analyzed. We found that knockdown of MeCP2 increased mRNA expression levels of epithelial markers E-Cadherin, and decreased mesenchymal markers, ZEB1, ZEB2, and TWIST1 in LN18 and U251 glioma cells (Fig. 3a-b).

**Fig. 4:**
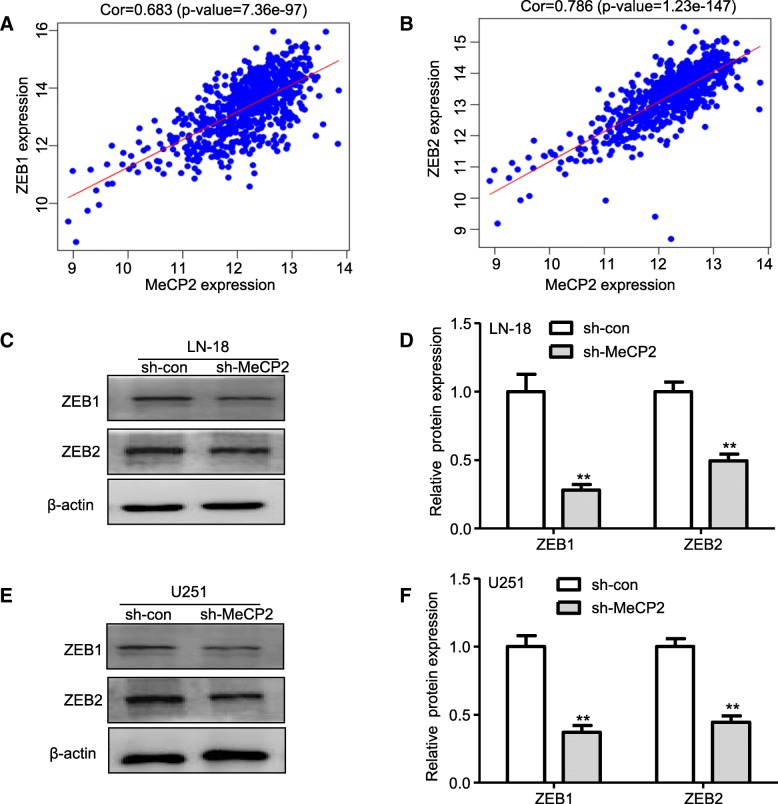
MeCP2 was correlated with EMT genes in glioma. **a**-**b** The correlation between MeCP2 mRNA levels, and ZEB1 and ZEB2 mRNA levels in glioma tissues was measured according to the TCGA cohort. **c**-**d** The levels of mRNA EMT genes in LN-18 glioma cells transfected with MeCP2 shRNA. **p < 0.01 vs. sh-con. **e**-**f** The levels of mRNA EMT genes in U251 glioma cells transfected with MeCP2 shRNA. **p < 0.01 vs. sh-con

Because MeCP2 could upregulate ZEB1, ZEB2, TWIST1 and E-Cadherin, we next examined whether MeCP2 is coexpressed with ZEB1, ZEB2, TWIST1 and E-Cadherin in human glioma samples. TCGA database analysis showed that ZEB1, ZEB2 and TWIST1 expression (Fig. 3c-e), but not E-Cadherin (Additional file 3: Fig. S1d), were increased in glioma. As shown in Fig. 3f-g, expression of ZEB1 and ZEB2 was significantly increased in glioma tissues compared with normal tissues. In addition, MeCP2 expression was positively correlated with ZEB1 and ZEB2 expression in the TCGA cohort (Fig. 4a-b), but not correlated with TWIST1 and E-Cadherin (Additional file 3: Fig. S1). When measured by western blotting, the expression of ZEB1 and ZEB2 protein was reduced in both LN18 and U251 glioma cells with knockdown of MeCP2 (Fig. 4c-f). All these results indicate a potent role for MeCP2 in the acquisition of mesenchymal phenotype in gliomas.

### MeCP2 knockdown suppresses EMT phenotype in a mouse xenograft model

**Fig. 5:**
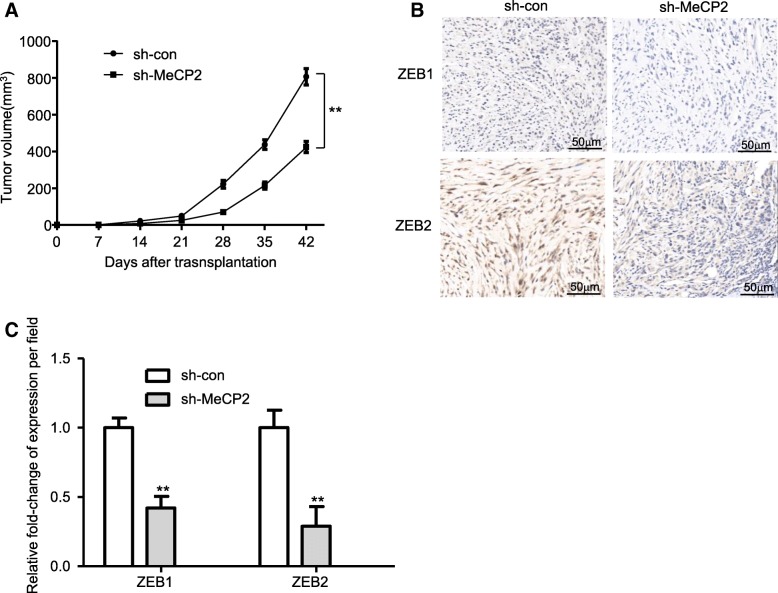
MeCP2 knockdown inhibited glioma cell EMT phenotype in vivo. **a** Size of tumors were measured every 7 days after injection. **p < 0.01 vs. sh-con. **b** Expression of ZEB1 and ZEB2 by staining immunohistochemical (IHC) in subcutaneous tumors of mice injected with sh-con or sh-MeCP2 cells. **c** The ZEB1-expressing cells and the ZEB2-expressing cells were qualified. **p < 0.01 vs. sh-con

To determine the potential impact of MeCP2 expression on EMT in glioma, a xenograft model was used. Sh-MeCP2 resulted in a significant growth reduction compared with sh-con (Fig. 5a). Moreover, ZEB1 and ZEB2 staining were used to detect the EMT in xenografted tumor tissues. As shown in Fig. 5b-c, knockdown of MeCP2 had fewer ZEB1 and ZEB2 positive cells compared with sh-con cells. These data suggested that MeCP2 knockdown in vivo functions similarly as in vitro.

### MeCP2 represses miR-200c in glioma

**Fig. 6:**
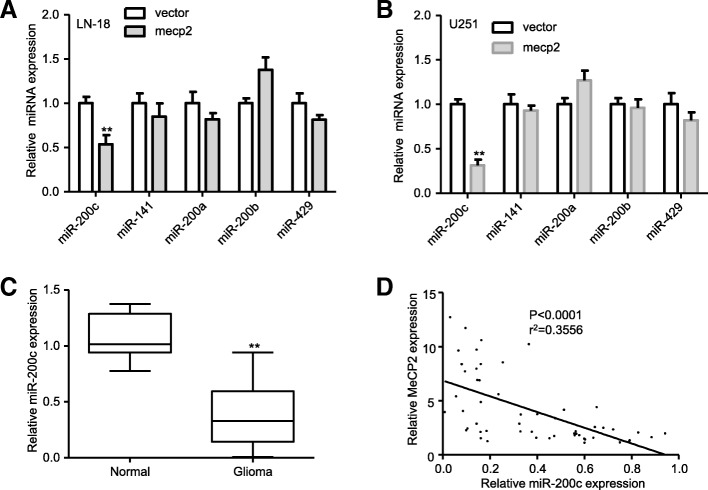
MeCP2 regulated miR-200 s in glioma. **a**-**b** The levels of miR-200 s expression in glioma cells transfected with MeCP2 plasmid. **p < 0.01 vs. vector. **c** The levels of miR-200c expression in normal brain and glioma tissues. **p < 0.01 vs. Normal. **d** The correlation between MeCP2 and miR-200c level was measured in glioma tissues

The miR-200 family can directly target ZEB1 and ZEB2, which served as a pivotal mediator of the EMT process [30]. Therefore, we postulate that MeCP2 may regulate EMT process by miR-200 family. As shown in Fig. 6a-b, expression of miR-200c, but not the other members of miR-200 family, was markedly reduced in MeCP2 overexpression glioma cells compared with vector. Additionally, MeCP2 knockdown induced miR-200c expression in glioma cells (Additional file 4: Fig. S2a). To investigate whether a relationship exists between MeCP2 expression and miR-200c, miR-200c expression was examined in human glioma samples. The downregulation of miR-200c expression was observed in glioma samples in comparison with normal brain tissues (Fig. 6c). To explore whether expression of miR-200c was related to MeCP2, the expression levels of MeCP2 and miR-200c were analyzed in glioma tissues. The correlation of high MeCP2 expression with low miR-200c expression in glioma tissues supports our finding that MeCP2 can downregulate miR-200c in glioma cells (Fig. 6d).

### miR-200c is involved in MeCP2 mediated-EMT in glioma

**Fig. 7:**
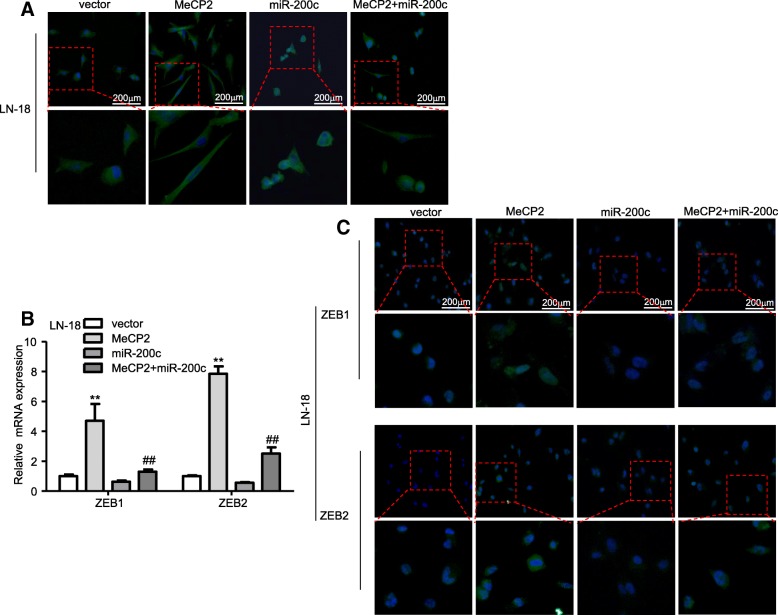
miR-200c alleviated LN-18 glioma cell EMT phenotype mediated by MeCP2. **a** Representative confocal images of cell morphology were captured after co-transfection with MeCP2 plasmid and miR-200c mimic. Green, β-tubulin. Blue, DAPI for nucleus. **b** The mRNA levels of ZEB1 and ZEB2 in glioma cells co-transfected with MeCP2 plasmid and miR-200c mimic. **p < 0.01 vs. vector; ^##^*P* < 0.01 vs. MeCP2. **c** Immunofluorescence staining was performed to assess the protein level of ZEB1 and ZEB2 expression in glioma cells co-transfected with MeCP2 plasmid and miR-200c mimic

To explore whether MeCP2 exerts biological functions via miR-200c, a rescue experiment was performed. As shown in Fig. 7a and Additional file 4: Fig. S2b, MeCP2 overexpression increased spindle shaped morphology, whereas miR-200c markedly reduced spindle shaped morphology induced by MeCP2. Because ZEB1 and ZEB2 is target genes of miR-200c, we wondered whether MeCP2 could modulate ZEB1 and ZEB2 by miR-200c. Ectopic expression of MeCP2 increased ZEB1 and ZEB2 mRNA expression, whereas miR-200c abrogated this increase (Fig. 7b and Additional file 4: Fig. S2c). Additionally, ectopic expression of MeCP2 increased positive ZEB1 and ZEB2 staining, miR-200c abrogated this increase (Fig. 7c and Additional file 4: Fig. S2d). These results may imply that MeCP2 regulate EMT of glioma in part by miR-200c.

### MeCP2 as a regulator of miR-200c transcriptional repression

**Fig. 8:**
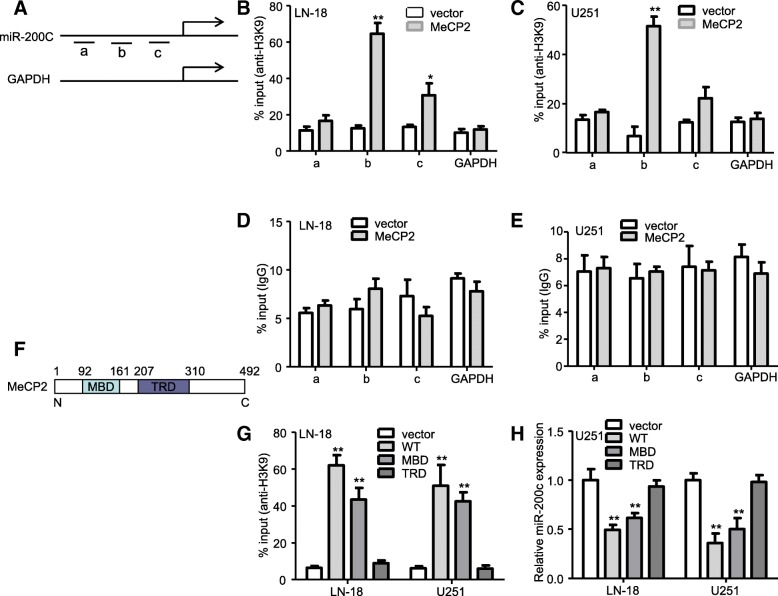
MeCP2 affected H3K9 occupancy at the miR-200c promoter in glioma cells. **a**-**e** ChIP analyses of MeCP2-over-expressing glioma cells were performed on GAPDH and miR-200c (primer set a: chr12:7071420–7,071,439; b:chr12:7072194–7,072,213;c:chr12:7072454–7,072,474) promoter regions by the indicated antibodies. Input as internal quality control for enrichment analysis. *P < 0.05, **P < 0.01 vs. vector. **f**-**g** ChIP was performed to analyze H3K9 occupancy at the miR-200c promoter in glioma cells transfected with MeCP2-WT, MeCP2-MBD and MeCP2-TRD. **P < 0.01 vs. vector. **h** expression of miR-200c was examined in glioma cells transfected with MeCP2-WT, MeCP2-MBD and MeCP2-TRD. **P < 0.01 vs. vector

MeCP2 can mediate transcriptional repression of genes by binding specifically to methylated DNA of genes [31]. To explore the mechanism how MeCP2 repress miR-200c expression in a similar manner, we predicted CpG of miR-200c promoter via using MethPrimer. Pyrosequencing results showed that methylation of miR-200c promoter was not significant change in MeCP2 overexpression compared with vector (Additional file 5: Fig. S3a). Previous study showed that MeCP2 can recruit histone methyltransferase that methylate local H3 lysine 9 (H3K9) are involved in gene silencing [32]. To explore whether MeCP2 represses the level of miR-200c expression via H3K9me3 modification in glioma cells, and the results found that a significant enrichment of H3K9me3 in the miR-200c promoter was observed in MeCP2-overexpressed glioma cells compared with vector cells (Fig. 8a-c). As expected, no alteration in IgG was observed in the miR-200c promoter region (Fig. 8d-e).

To understand how MeCP2 regulates H3K9me3 enrichment in the promoter of miR-200c, we constructed WT, MBD and TRD plasmids to transfect into glioma cells. We found that in lysates of glioma cells, H3K9me3 enrichment in the promoter of miR-200c was significantly increased in MBD and MeCP2 group, but not TRD, compared with vector group (Fig. 8f-g). In addition, we found that miR-200c expression was markedly reduced by expression of either WT or MBD truncated form of MeCP2 (Fig. 8h), suggesting that MBD is capable of exerting transcriptional repression activity as WT MeCP2.

### MeCP2 mediated H3K9me3 of miR-200c via its interaction with SUV39H1

**Fig. 9:**
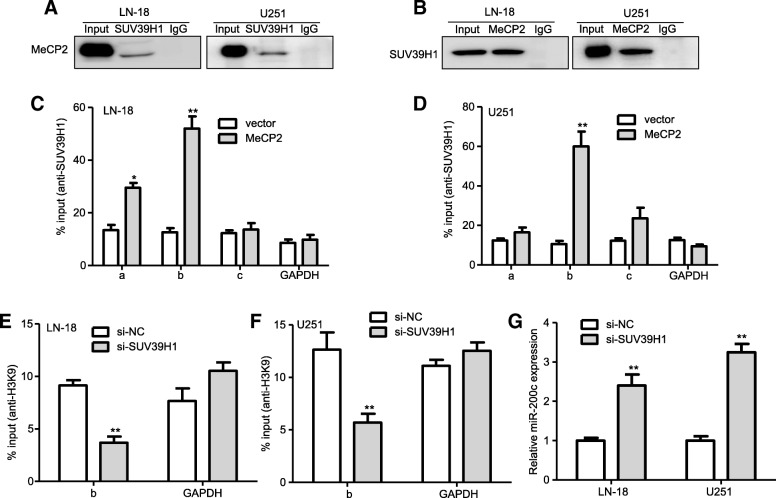
The interaction of suv39h1 with MeCP2 mediated miR-200c. **a**-**b** Endogenous MeCP2 co-immunoprecipitated with endogenous suv39h1 was observed by co-immunoprecipitation analysis. **c**-**d** ChIP-PCR shows that overexpression of MeCP2 led to an increase of suv39h1 bound to miR-200c. *P < 0.05, **P < 0.01 vs. vector. **e**-**f** ChIP was performed to analyze H3K9 occupancy at the miR-200c promoter in glioma cells transfected with si-NC and si-Suv39h1. **P < 0.01 vs. vector. **g** expression of miR-200c was examined in glioma cells transfected with si-NC and si-Suv39h1. **P < 0.01 vs. vector

MeCP2 initiates silencing with selective methylation on H3K9, thus creating a high-affinity binding site for suppressor of variegation 39H1(SUV39H1) proteins [33].To investigate the interaction between MeCP2 and SUV39H1, coimmunoprecipitation(Co-IP) experiments was used to perform in glioma cells. Endogenous MeCP2 coimmunoprecipitated with endogenous SUV39H1 in glioma cells (Fig. 9a). The interaction of SUV39H1 with MeCP2 was further confirmed via reverse endogenous coimmunoprecipitation of SUV39H1 with MeCP2 (Fig. 9b), supporting a physical SUV39H1-MeCP2 interaction in vitro. These data prompted us to investigate whether SUV39H1 is involved in H3K9me3 of miR-200c mediated by MeCP2, ChIP analysis was performed in MeCP2-overexpressing glioma cells. We found that MeCP2 enhanced the binding of SUV39H1 to the promoters of miR-200c (Fig. 9c-d). However, no increase in SUV39H1 binding to the promoters of GAPDH, a gene not mediated by SUV39H1, was observed (Fig. 9c-d). These results indicated that MeCP2 is related to SUV39H1 occupancy at miR-200c promoter. To further confirm whether SUV39H1 mediated epigenetic repression of miR-200c, we examined miR-200c expression after transfection of si-SUV39H1 to glioma cells (Additional file 5: Fig. S3b). As shown Fig. 9e-g, SUV39H1 knockdown reduced H3K9me3 occupancy at the promoter of miR-200c and resulted in the increase of miR-200c expression. Collectively, these results revealed that SUV39H1 might be implicated in the function of MeCP2 as an epigenetic repressor of miR-200c in glioma cells.

## Discussion

Emerging evidences indicate MeCP2 as a pivotal oncogene in tumorigenesis. MeCP2 as a frequently amplified oncogene has observed in cancer [34–36]. Recently, overexpression of MeCP2 have found in diverse types of cancers, including gastric cancer, endometrial cancers, and prostate cancer, whereas MeCP2 knockdown repressed the proliferation of tumor cells [35, 37, 38]. However, the role of MeCP2 in glioma is still largely unknown. Here, we showed that the expression of MeCP2 was increased in glioma tissues and associated with pathological grade. Interestingly, MeCP2 expression are highly in mesenchymal GBM subtype, suggesting that MeCP2 may be involve in EMT of glioma. Recently, Li et al. found that MeCP2 promoted EMT by epigenetically silencing BMP7 in endothelial cells [39]. Here, we found that knockdown of MeCP2 significantly induced epithelial phenotype in vitro, whereas overexpression of MeCP2 exerted the opposite effect. These results indicate that MeCP2 may play a key role in the regulation of EMT in gliomas.

EMT is a cellular mechanism that is known to promote a developmental transdifferentiation program [40]. In addition, during EMT, epithelial cells lose their polarity, which will increase express mesenchymal markers, such as ZEB1 and ZEB2, and then leading to acquire invasive potential [41, 42]. This phenomenon has also been related to tumor progression, during which epithelial cells lose signs of differentiation, and obtain enhanced migratory abilities, which results in invasion and metastasis [43, 44]. ZEB1 increases loss of cell–cell contact and therefore fosters increased motility in glioma [45]. Additionally, ZEB2 knockdown inhibited proliferation, migration, invasion, and increased cell death in glioma-derived cell cultures [46]. Here, we found that inhibition of MeCP2 markedly impaired expression of the EMT player ZEB1 and ZEB2 in vitro and in vivo. These results indicated that MeCP2 can function as a transcriptional activator of ZEB1 and ZEB2 in glioma. In this paper, though some important discoveries were disclosed, a subcutaneous tumor model was used in our study, which may result in the consequences that we may not be able to perfectly simulate the microenvironment of intracranial tumor growth in vivo.

Previous study showed that MeCP2 is best known for its role as a transcriptional repressor in the regulation of gene expression in mammalian cells. MeCP2 was associated with the methylated of TFPI-2 promoter, and the loss of MeCP2 in TFPI-2 promoter resulted in gene reactivation in human glioma cells [47]. Recently, it was found that dysregulation of miRNA in cancer is due to the transcriptional repression function of MeCP2 [48, 49]. MiR-200 s family have been reported to play a central role in suppressing EMT marker ZEB1 and ZEB2. Therefore, we speculate that MeCP2 may mediate a transcriptional activator of ZEB1 and ZEB2 by repressing miR-200 s family. Here, we showed that MeCP2 repressed expression of miR-200c, but not other member of miR-200 family, in glioma cells, and MeCP2 expression was negatively associated with miR-200c in glioma tissues. In addition, miR-200c was involved in MeCP2-mediated EMT in glioma. These results suggest that MeCP2 may regulate EMT, at least in part, by miR-200c in glioma.

MeCP2 is believed to exert the function of transcriptional inhibition via binding to methylated CpG dinucleotides [50]. Unexpectedly, we found that overexpression of MeCP2 not changed the methylation of miR-200c promoter. As a master regulator of gene expression, MeCP2 also acts as a transcriptional repressor by recruiting corepressors and epigenetic regulator, to the promoter regions to suppress a variety of genes expression [51]. The association of MeCP2 with H3K9 methylation has been reported in the region of IL-6 gene [32]. A direct correlation between the binding of MeCP2 and the presence of H3K9me in promoter of the IκBα gene and the BDNF gene also has been described [52, 53]. Our ChIP results showed, for the first time, that MeCP2 represses expression of miR-200c by regulating H3K9 methylation of miR-200c promoter. The association of MeCP2 with histone H3 methyltransferase activity is primarily mediated by methyl-CpG-binding domain (MBD), represents a specific epigenetic mark for transcriptional repression [51].

Here, we showed that MeCP2 mainly exhibit transcriptional repression of miR-200c in glioma depends on its MBD domain, but not TRD domain. As an oncogene, whether MeCP2 exerts its effect in the control of glioma by interacting with other miRNA is still unclear, which is the field we will focus on in subsequent study.

Lunyak et al. reported that MeCP2 initiates silencing with selective methylation on H3K9 via interactions with SUV39H1 proteins [33].The molecular mechanism underlying its role in epigenetic regulation is yet unknown. Here, we showed that MeCP2 interacts with SUV39H1 to mediate methylation-based epigenetic transcriptional silencing. Our observation showed that MeCP2 is associated with SUV39H1, and aids in the recruitment of SUV39H1 to the promoter. Then, SUV39H1 recruitment induced in the accumulation of H3K9, which represses miR-200c expression at the transcriptional levels. In consistent with the previous study of MeCP2 interacted with SUV39H1 to regulate their target genes suggests that this pattern of mechanism may be more general.

## Conclusions

**Fig. 10:**
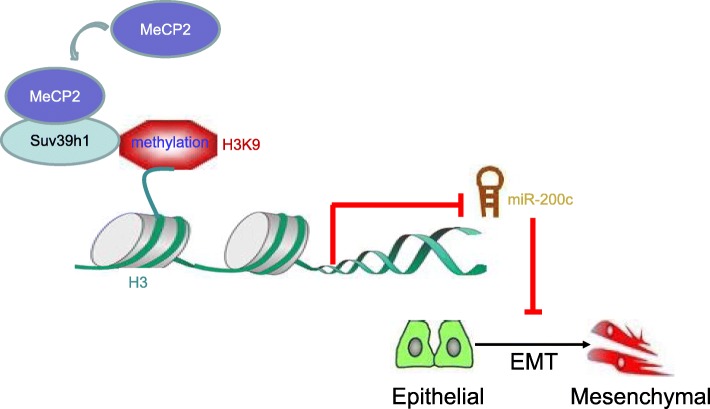
Schematic diagram showing the mechanism of the interaction of MeCP2 with SUV39H1, and increased the accumulation of the repressive marks H3K9 in the promoter of miR-200c, resulting in the transcriptional repression of the miR-200c, which induced EMT in glioma

In summary, our findings demonstrated that the interaction of MeCP2 with SUV39H1, and deliver H3K9 to the promoter of miR-200c, resulting in the transcriptional repression of the miR-200c, and then activates EMT in glioma (Fig. 10). These results suggest that MeCP2 as an attractive therapeutic target, the inhibition of which may potentially downregulate the expression of miR-200c, resulting in subsequent suppression of the EMT. Hence, our study not only reveals a novel mechanism underlying the epigenetic regulation of EMT in glioma but also provides a novel therapeutic target of glioma. Moreover, identification of MeCP2-specific inhibitor could potentially create a new paradigm in the discovery and development of molecular target therapy for glioma.

## Additional files

Additional file 1: **Table S1.** The clinicopathological features of glioma patients. (DOC 32 kb)

Additional file 2: **Table S2.** Primers used for qPCR, ChIP, Pyrosequencing Assays. (DOC 214 kb)

Additional file 3: **Figure S1.** (A) U251 glioma cell morphology was captured with optical microscope after transfection with MeCP2 shRNA for 2 weeks. (B) The levels of MeCP2 mRNA expression were examined after transfection with MeCP2 plasmid. **p < 0.01 vs. vector. (C) Representative images of U251 cell morphology were captured after transfection with MeCP2 plasmid. Green, β-tubulin. Blue, DAPI for nucleus. (D)The level of E-Cadherin in glioma and normal tissues samples from TCGA cohort. Red, tumor. Black, normal. (E-F) The correlation between MeCP2, and E-Cadherin and TWIST1 in glioma tissues was measured according to the TCGA cohort. (DOCX 282 kb)

Additional file 4: **Figure S2.** (A) The levels of miR-200c in glioma cells transfected with MeCP2 shRNA. **p < 0.01 vs. sh-con. (B) Representative confocal images of U251 glioma cell morphology were captured after co-transfection with MeCP2 plasmid and miR-200c mimic. Green, β-tubulin. Blue, DAPI for nucleus. (C) The mRNA levels of ZEB1 and ZEB2 in U251 glioma cells co-transfected with MeCP2 plasmid and miR-200c mimic. **p < 0.01 vs. vector; ^##^*P* < 0.01 vs. MeCP2. (D) Immunofluorescence staining was performed to assess the protein level of ZEB1 and ZEB2 expression in U251 glioma cells co-transfected with MeCP2 plasmid and miR-200c mimic. (DOCX 279 kb)

Additional file 5: **Figure S3.** (A) The methylation of miR-200c promoter was observed after transfection with MeCP2 plasmid. (B) The levels of SUV39H1 mRNA expression were examined after transfection with si-SUV39H1. **p<0.01 vs. sh-con. (DOCX 58 kb)

## Data Availability

Additional data are available as Supplementary information.

## Abbreviations

ChIP: Chromatin immunoprecipitation
Co-IP: Co-immunoprecipitation
EMT: Epithelial-to-mesenchymal transition
HDAC: Histone deacetylase complex
MBD: Methyl-CpG-binding domain
MECP2: Methyl CpG-binding protein 2
miRNAs: MicroRNAs
NHA: Normal human astrocytes
RTT: Rett syndrome
SUV39H1: Suppressor of variegation 39H1
TRD: Transcriptional repression domain
