# Supplementary material for: A central role for MeCP2 in the epigenetic repression of miR-200c during epithelial-to-mesenchymal transition of glioma
Source: J Exp Clin Cancer Res. 2019 Aug 20;38:366. doi: 10.1186/s13046-019-1341-6 (PMC6702741; doi:10.1186/s13046-019-1341-6)
Supplement: Supplementary file 1 — Table S1. The clinicopathological features of glioma patients. (DOC 32 kb) [file 13046_2019_1341_MOESM1_ESM.doc]

**TABLE 1. The clinicopathological features of glioma patients**

**Parameters Total**

Age(years)

<50 21

>50 44

Gender

Male 31

Female 34

Clinical grade

Low grade Ⅰ-Ⅱ 22

High grade Ⅲ-Ⅳ 43

KPS

>80 47

<80 18
