# Supplementary material for: A central role for MeCP2 in the epigenetic repression of miR-200c during epithelial-to-mesenchymal transition of glioma
Source: J Exp Clin Cancer Res. 2019 Aug 20;38:366. doi: 10.1186/s13046-019-1341-6 (PMC6702741; doi:10.1186/s13046-019-1341-6)
Supplement: Supplementary file 2 — Table S2. Primers used for qPCR, ChIP, Pyrosequencing Assays. (DOC 214 kb) [file 13046_2019_1341_MOESM2_ESM.doc]

**Table S1. Primers used for qPCR, ChIP, Pyrosequencing Assays**

| **Target** | **Primer name** | | **Sequence** |
| --- | --- | --- | --- |
| **qPCR** | | | |
| MeCP2 | | MeCP2 _F | GCCGAGAGCTATGGACAGCA |
|  |  | MeCP2_R | CCAACCTCAGACAGGTTTCCAG |
| ZEB1 | | ZEB1_F | ACTCTGATTCTACACCGC |
|  |  | ZEB1_R | TGTCACATTGATAGGGCTT |
| ZEB2 | | ZEB2_F | TGAGGATGACGGTATTGC |
|  |  | ZEB2_R | ATCTCGTTGTTGTGCCAG |
| TWIST1 | | TWIST1_F | GTCCGCAGTCTTACGAGGAG |
|  |  | TWIST1_R | TGGAGGACCTGGTAGAGGAA |
| E-Cadherin | | E-Cadherin _F | GCCCCATCAGGCCTCCGTTT |
|  |  | E-Cadherin _R | ACCTTGCCTTCTTTGTCTTTGTTGGA |
| vimentin | | vimentin _F | CCTGAACCTGAGGGAAACTAA |
|  |  | vimentin _R | GCAGAAAGGCACTTGAAAGC |
| N-cadherin | | N-cadherin _F | TGGACCATCACTCGGCTTA |
|  |  | N-cadherin _R | TGGACCATCACTCGGCTTA |
| ZO-1 | | ZO-1 _F | CACGCAGTTACGAGCAAG |
|  |  | ZO-1 _R | CACGCAGTTACGAGCAAG |
| SUV39H1 | | SUV39H1_F | CATAGACAACCTTGATGAGCG |
|  |  | SUV39H1_R | GCAGGATTCAGTCCCACAC |
| GAPDH | | GAPDH _F | AGCAAGAGCACAAGAGGAAG |
|  |  | GAPDH _R | GGTTGAGCACAGGGTACTTT |
| **ChIP** | | | |
| Promoter  miR-200c | Promoter-miR-200c-a _F | | GTCACAGGCATTCACAGTCC |
|  | Promoter-miR-200c-a _R | | TCCCAGGCTTTGTTTGCTTC |
| Promoter miR-200c -b | Promoter-miR-200c-b _F | | GCAGGTTGGAGAAAAGAGGC |
|  | Promoter-miR-200c-b _R | | CTTAACCCCTTCCCTCCCAG |
| Promoter miR-200c-c | Promoter-miR-200c-c _F | | TTAAAGCCCCTTCGTCTCCC |
|  | Promoter-miR-200c-c _R | | CGACACACACCGATTTACCC |
| Promoter GAPDH | Promoter- GAPDH_F | | TACTAGCGGTTT TACGGGCG |
|  | Promoter- GAPDH_R | | TCGAACAGGAGGAGCAGAGAGCGA |
| **Pyrosequencing Assays** | | | |
| miR-200c  (Prom 1) | Forward | | GGTTGAGTTTGGGATTGTAGAG |
|  | Reverse | | AAACCCAAATTACAATCCAAACAAAC |
|  | Sequencing | | GATGAGGGTGGGTAA |
| miR-200c  (Prom 2) | Forward | | AGGGTTTGTTTGGATTGTAAT |
|  | Reverse | | CCCACCTTAAATCAAACAACTT |
|  | Sequencing | | GGTTTGTTTGGATTGTAATT |
| miR-200c  (Prom 3) | Forward | | ATTTTGGGTTTGAAGTTGTTTGA |
|  | Reverse | | CACAAAAACAAAAACCTCCATCATTAC |
|  | Sequencing | | TGAAGTTGTTTGATTTAAGG |
