# Supplementary figures and images for: A central role for MeCP2 in the epigenetic repression of miR-200c during epithelial-to-mesenchymal transition of glioma
Source: J Exp Clin Cancer Res. 2019 Aug 20;38:366. doi: 10.1186/s13046-019-1341-6 (PMC6702741; doi:10.1186/s13046-019-1341-6)

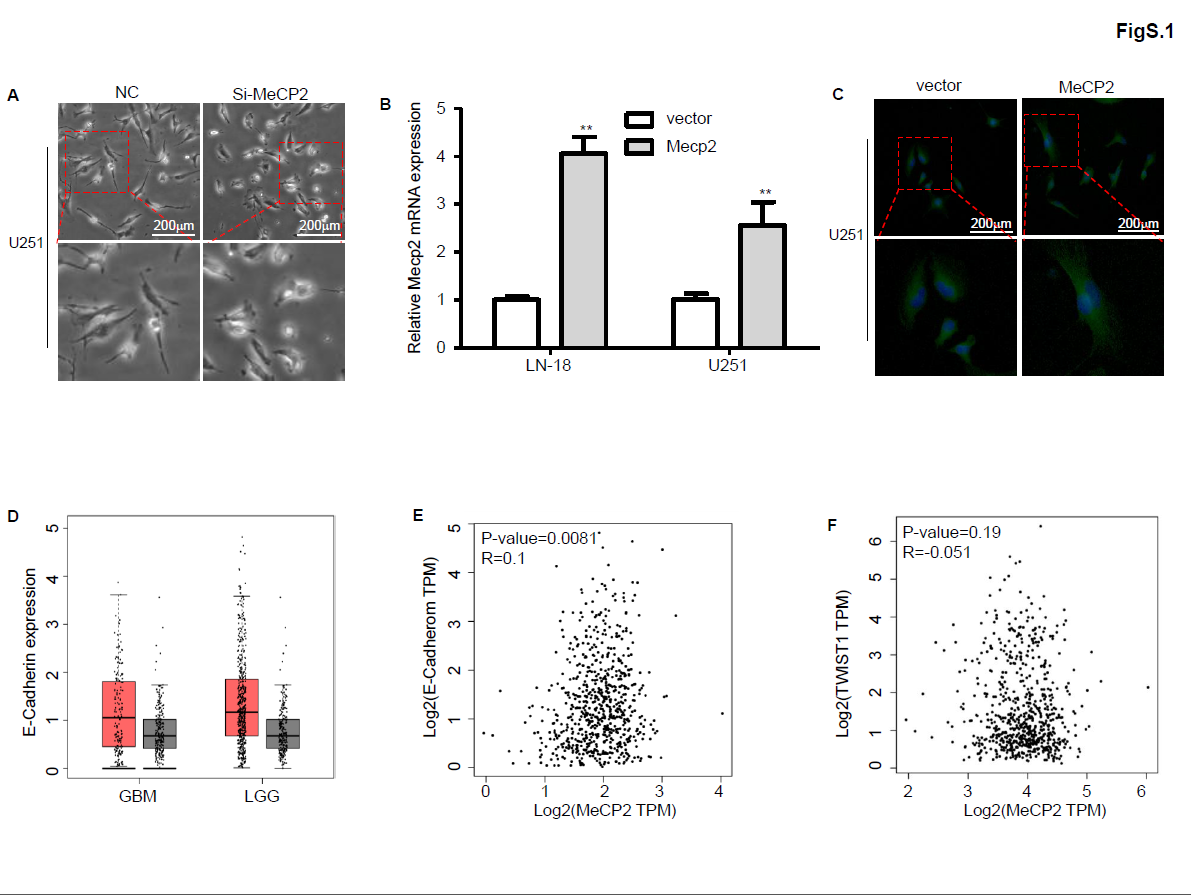

Supplement: Supplementary file 3 — Figure S1. (A) U251 glioma cell morphology was captured with optical microscope after transfection with MeCP2 shRNA for 2 weeks. (B) The levels of MeCP2 mRNA expression were examined after transfection with MeCP2 plasmid. **p < 0.01 vs. vector. (C) Representative images of U251 cell morphology were captured after transfection with MeCP2 plasmid. Green, β-tubulin. Blue, DAPI for nucleus. (D)The level of E-Cadherin in glioma and normal tissues samples from TCGA cohort. Red, tumor. Black, normal. (E-F) The correlation between MeCP2, and E-Cadherin and TWIST1 in glioma tissues was measured according to the TCGA cohort. (DOCX 282 kb) [file 13046_2019_1341_MOESM3_ESM.docx]

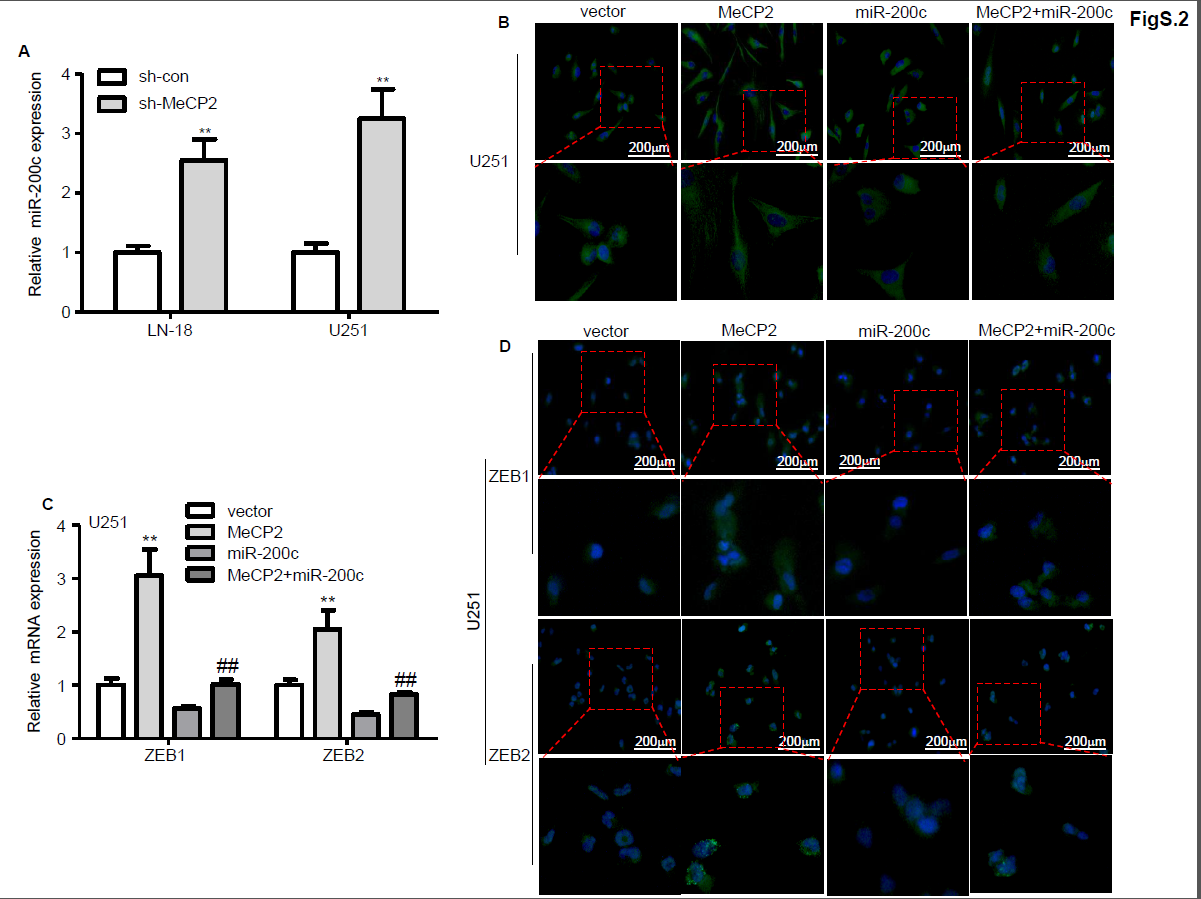

Supplement: Supplementary file 4 — Figure S2. (A) The levels of miR-200c in glioma cells transfected with MeCP2 shRNA. **p < 0.01 vs. sh-con. (B) Representative confocal images of U251 glioma cell morphology were captured after co-transfection with MeCP2 plasmid and miR-200c mimic. Green, β-tubulin. Blue, DAPI for nucleus. (C) The mRNA levels of ZEB1 and ZEB2 in U251 glioma cells co-transfected with MeCP2 plasmid and miR-200c mimic. **p < 0.01 vs. vector; ##P < 0.01 vs. MeCP2. (D) Immunofluorescence staining was performed to assess the protein level of ZEB1 and ZEB2 expression in U251 glioma cells co-transfected with MeCP2 plasmid and miR-200c mimic. (DOCX 279 kb) [file 13046_2019_1341_MOESM4_ESM.docx]

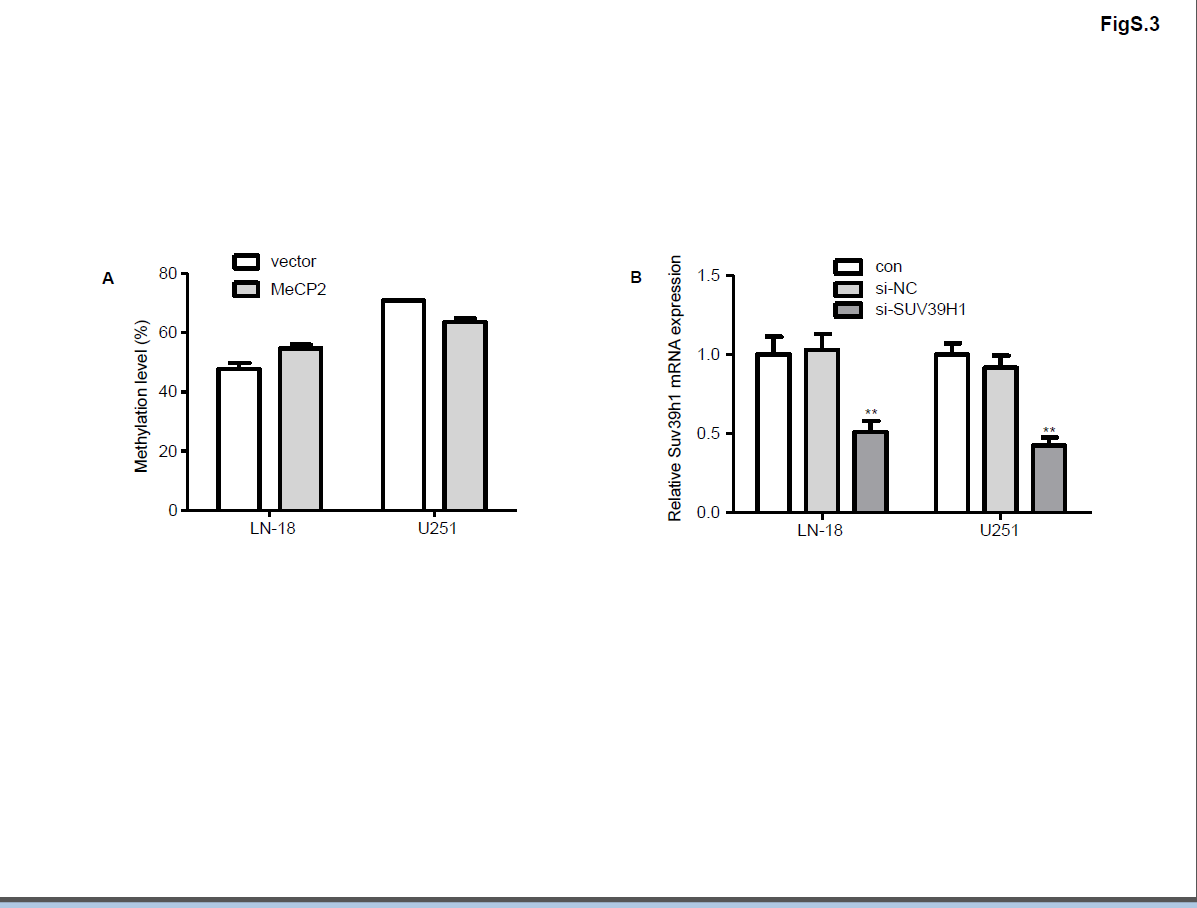

Supplement: Supplementary file 5 — Figure S3. (A) The methylation of miR-200c promoter was observed after transfection with MeCP2 plasmid. (B) The levels of SUV39H1 mRNA expression were examined after transfection with si-SUV39H1. **p<0.01 vs. sh-con. (DOCX 58 kb) [file 13046_2019_1341_MOESM5_ESM.docx]
